# A method to extract fishers’ knowledge (FK) to generate evidence for sustainable management of fishing gears

**DOI:** 10.1016/j.mex.2019.05.008

**Published:** 2019-05-08

**Authors:** Paritosh C. Deshpande, Helge Brattebø, Annik Magerholm Fet

**Affiliations:** a Department of Industrial Economics and Technology Management, Norwegian University of Technology, NO-7491 Trondheim, Norway; b Department of Energy and Process Engineering, Industrial Ecology Programme, Norwegian University of Technology, NO-7491 Trondheim, Norway

**Keywords:** ALDFG, abandoned, lost and discarded fishing gears, FG, fishing gears, FK, fishers’ knowledge, LEK, Local Ecological Knowledge, MFA, material flow analysis, MoP, Mass of Plastic, Design protocol of survey based questionnaire, Fishers’ knowledge, Survey, Fishing gears, Marine pollution, Resource management, Delphi method, Questionnaire

## Abstract

The dangerous effects of Abandoned, Lost or Discarded Fishing Gears (ALDFG) is documented in the literature. However, there exists an overall lack of understanding in quantifying the pollution loads of fishing gears (FG) in territorial waters or on the beaches. The lack of data on FG life cycle results in mismanagement of one of the troublesome resources across the globe. In the remote and data-less situations, local stakeholders’ knowledge remains the only source of information. Therefore, in this article, we propose:

• A methodology to extract fishers’ knowledge (FK) for generating evidence on FG handling and management practices in Norway.
• The stepwise approach includes mapping of relevant stakeholders, drafting and finalizing a structured questionnaire using the Delphi method among experts to build the consensus and finally, statistically analyzing the recorded responses from the fishers.
• The questions are designed to extract both qualitative and quantitative information on purchase, repair, gear loss and disposal rates of commercial FGs.

The responses from 114 Norwegian fishers are recorded, analyzed and presented as a part of method validation.

The evidence from the survey is then used as an input to coin the regional FG handling and management strategies in Norway. The presented method is proven a robust strategy to retrieve scientific information from the local stakeholders’ and can easily be replicated elsewhere to build global evidence around the ALDFG problematic.

## Graphical abstract

**Figure:**
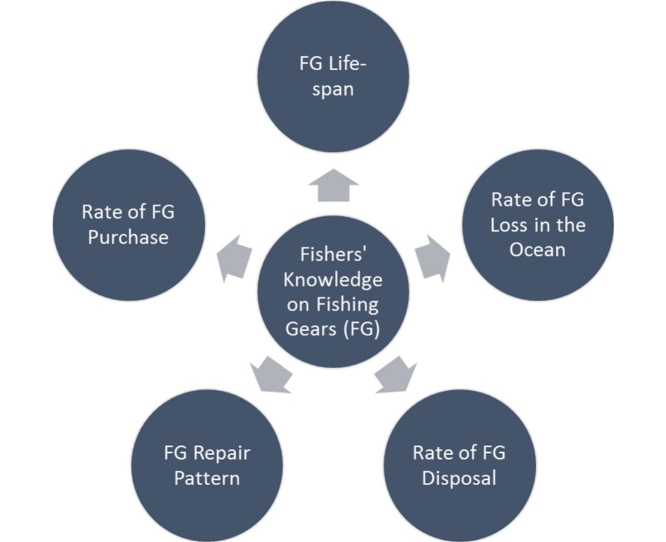

**Specifications Table**

**Table:** 

Subject Area:	*Environmental Science* *Social Sciences*
More specific subject area:	*Environmental monitoring, Resource management*
Method name:	*Design protocol of survey based questionnaire.*
Name and reference of original method:	The applied method in this study is a stepwise framework used to extract quantitative and qualitative information. The required information is collected from fishers through structured questionnaire designed using Delphi method. The article presents the modification of methods discussed in:
	Johannes, R. E., Freeman, M. M. R. & Hamilton, R. J. 2000. Ignore fishers' knowledge and miss the boat. *Fish and Fisheries,* 1, 257-271.
	Leite, M. C. F. & Gasalla, M. A. 2013. A method for assessing fishers’ ecological knowledge as a practical tool for ecosystem-based fisheries management: Seeking consensus in Southeastern Brazil. *Fisheries Research,* 145, 43-53.
Resource availability:	One Supplementary Information file is provided with this manuscript: 1 SI-1: Sample survey questions (English version) used to conduct fishers survey.

## Method details

Past two decades observed significant surge on adopting Local Ecological Knowledge (LEK) in the mainstream research areas of natural resource conservation and sustainable resource management. LEK refers to a body of knowledge accumulated over time and transformed into an individual’s perception of the resource, which is then presented as the communities’ collective knowledge [1]. It is often based on long-term observations of the local ecosystem considering local variations, behavioral patterns and focusing on essential resources/species of the concerned ecosystem [2]. Practical applications of LEK ranges from a variety of systems including, but not limited to, small-scale agriculture, horticulture, forestry and fisheries [3]. Johannes [4] and colleagues played a key role in establishing and documenting the use of LEK in the sector of fishery management through their work between 1980 and 2000. In his first documented study on applying fishers’ knowledge (FK), Johannes [5] emphasized the variety and depth of information local fishers’ possess on marine ecology and conservation, fish behavior/habitats, fishing practices, fishing gear types and other ecosystem concepts. Further, Johannes et al. [6] argued that by ignoring such readily available and inexpensive source of knowledge while studying the local system, humanity runs the danger of “missing the boat” on fisheries sustainability. Although fishers possess a valuable source of information, integrating or translating that information to the science of resource management demands creativity in applying suitable scientific methods [3,7]. So far, application of FK was demonstrated to manage biodiversity and marine protected areas [5,8], studying fish species, habitats and catch patterns [9,10], fishery resource management [2,3,11] and to understand the impacts of fishing methods and equipment [12,13]. In this study, we present a stepwise method to extract FK on fishing gear (FG) use and handling practices in Norway.

In commercial fishing, FGs are one of the vital resources to fishers. Recent advancements in the gear design and technology allowed substantial growth in catch quantities in commercial fishery [14]. Improvements in gear design were initiated with the replacement of natural fibers such as jute, yarn, cotton with the synthetic fibers such as PP, PE, and Nylon. Although, unlike natural fibers, synthetic FGs are functionally resistant to degradation in the water, and, once discarded or lost, these gears may remain in the marine environment for decades as ghost FGs [30,15]. These ghost FG are considered as one of the deadliest fractions of marine waste with adverse impact on marine ecology and fishers economy [[15], [16], [17], [18], [19]], however, lack of quantitative information and evidence crippled the possibilities to make informed decisions on avoiding or minimizing the probabilities of gear loss upon deployment. Additionally, fraction of these lost nets drifts along the tidal currents and may end-up on the beaches or marshes causing land-pollution and pose entanglement risks to birds and marine animals. Furthermore, FGs lost in the ocean or on land is not only damaging to the environment but also a lost opportunity to recycle and reuse the resources.

To develop the management strategies for FG resources, it is essential to build the holistic understanding on typical life span, rates of gear loss, disposal, and repair patterns of commercial FGs. The stepwise framework proposed in this study is aimed at generating evidence to aid the sustainable management of FG resources in Norway.

### Commercial fishery of Norway

**Fig. 1:**
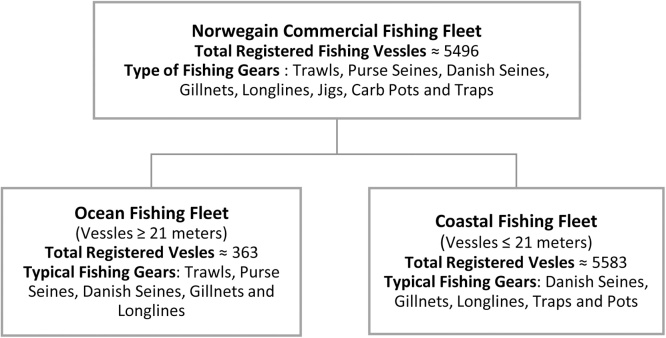
Structure of commercial fishing fleet of Norway [21].

Norway is a Northern European country surrounded by water to the south (Skagerak), the west (the North Sea and the Norwegian Sea), the north and north-east (the Barents Sea). With a marine resource-rich coastline of more than 25,000 km, Norway is the European leader regarding both capture fishery and aquaculture [20]. The capture fishery has always played a critical social and economic role, nationally and regionally, and has been the basis for settlement and employment along the entire Norwegian coast [14]. Commercial capture fishery sector is segmented into the coastal and ocean fishing fleet. The coastal fishing fleet comprises of smaller vessels manned by 1–5 fishers and size ranges from 10 to 20 meters. On the other hand, ocean fleet is known for its deep-water and sophisticated fishing practices, where fishing vessels are generally more than 28 m in size and crew members can vary from 20 persons or more [14,21]. In 2016, a total of 5946 vessels are registered in Norway out of which approximately 90% are coastal vessels, and the rest is ocean fishing fleets [21]. The primary capture species include herring, cod, capelin, mackerel, saithe, blue whiting, and haddock. A few additional species are caught in smaller quantities but have a high commercial value such as prawns, Greenland halibut, and ling. Fig. 1 shows the diversification of fishing fleet concerning the number of vessels, type of FGs they use.

## Material and methods

### Survey and questionnaire

Based on the literature on applications of FK in managing fishery resources, surveys in the form of questionnaire is considered to be an effective method to extract the information from local fishers [3,10,13,[22], [23], [24], [25]]. Accordingly, a systematic questionnaire, comprised of both qualitative and quantitative questions, is designed using the Delphi Method to reach the consensus on language, structure, and content of the questions. Delphi method involves applying rounds of consultations to a set of experts on a selected subject. After each round of consultation, the results of all the responses are summarized and presented individually to each participant. Participants can further change their opinion/views after the newly presented data and the similar rounds of consultation continues until one finds the consensus on the selected subject. The Delphi method is being practiced extensively in social sciences to find consensus, while a fundamental premise is the ability to maintain respondent anonymity throughout the process [23,26].

**Fig. 2:**
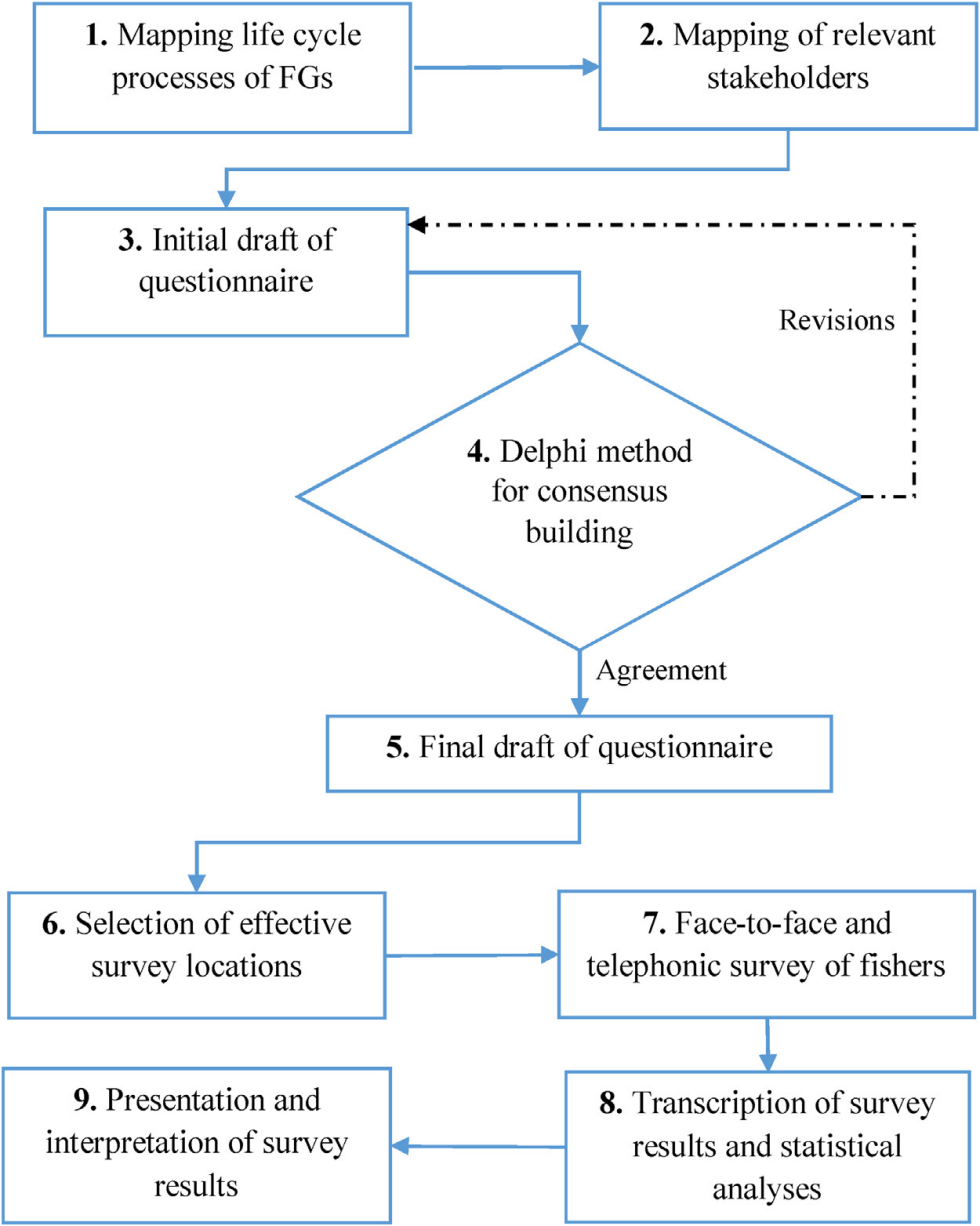
Proposed stepwise methodology to extract fishers’ knowledge using questionnaire.

Fig. 2 shows the systematic stepwise approach and an application of a Delphi method. First, a system life cycle processes of FGs and relevant key stakeholders are identified and presented in Deshpande and Aspen [27]. In the third step, a structured draft of the questionnaire was created to extract information on the handling and management of FGs throughout their life cycle. Six FG types commonly used by the Norwegian commercial fishers are selected for the study namely, Trawls, Danish seines, purse seines, gillnets, longline and traps/pot. The experts in the field of the fishery (Fishers Association), FG manufacturers, environment and resources consultants and researchers in marine and fishery sciences were contacted and were asked to evaluate and comment on the language, structure, and clarity of the designed questionnaire for fishers. The objective of the survey was to capture the pattern with which Norwegian fishers operate and manage FG types but also design a survey, quick to fill-up andcorrespondingto the fisher’s experiences. The consensus was reached after two rounds of revisions as per the Delphi method and a set of 13-questions, consisting of both qualitative and quantitative questions, were finalized covering the following topics:

• Norwegian fishers and fishing vessels
• Selected FG types owned by a fishing company
• Annual purchase patterns for new FGs
• Annual repair pattern and frequency of FGs
• The typical lifespan of selected FGs
• The average annual rate of FG loss in the ocean
• Waste management of FGs

After finalizing the set of questions, survey sites were chosen to interact with fishers. The collected responses are then transported as excel sheets and transfer coefficients are calculated based on the developed formula. Finally, all the results are presented, and interpreted to build the evidence on FG life cycle. The final questionnaire used for data collection is given in the supplementary material.

### Statistical analysis

To analyze the data quantitatively, the answers from survey responses were transcribed from the survey software, (e.g. SurveyMonkey), and distributed into answer groups. Quantitative data is treated to estimate the statistical inference, the sample mean, standard deviation and 95% confidence interval of the sample size. The rate of repair, a fraction of part replacement and typical life-span of FGs are estimated using basic statistical operations of a sample mean, standard deviation, and 95% confidence interval. Further, the transfer coefficients (TC) C_stock_, C_Lost_ and C_Disposal_ are calculated using the following formula.

1 C_stock_ represents the rate of an annual turnover of selected FGs for the sampled fishing companies in Norway. C_stock_ is a ratio of units of FGs available after the loss and disposal of FGs in a given year to the units of FGs purchased by a fishing company in a given year. Knowing the units/mass of FGs sold to the regional fishing fleet, this rate can be used to estimate total units/mass of selected FGs available at any point of time for a given region. In this study, we focused on estimating the mass of plastics (MoP) present in the stock of the Norwegian commercial fishing fleet.

$${C_{stock}}_{\left(a,b,c,d,e,f\right)}=\frac{\sum_{n=1}^{114}{\left(\frac{{FG}_{o}-{FG}_{L}-{FG}_{D}}{{FG}_{P}}\right)}_{\left(a,b,c,d,e,f\right)}}{N_{\left(a,b,c,d,e,f\right)}}$$

*Where, a = Trawls, b = Purse Seine, c = Danish Seines, d = Gillnets, e = Longlines, f = Traps/pots*

*FG_O_* = *Number of FGs owned by a fishing company*

*FG_L_* = *Number of FGs lost annually by a fishing company*

*FG_D_* = *Number of FGs disposed annually by a fishing company*

*FG_P_* = *Number of FGs purchased annually by a fishing company*

*N* = *Total number of responses for each FG type*

2 C_Lost_ represents the typical rate at which fishers lose their FGs in the ocean upon deployment in a given year and is calculated for each FG type as an average of the ratio of reported FGs lost by a fishing company to the total FGs owned by the fishing fleet.

$${C_{Lost}}_{\left(a,b,c,d,e,f\right)}=\frac{\sum_{n=1}^{114}{\left[\frac{{\left({FG}_{L}\right)}_{n}}{{\left({FG}_{O}\right)}_{n}}\right]}_{\left(a,b,c,d,e,f\right)}}{N_{\left(a,b,c,d,e,f\right)}}$$

*Where, a = Trawls*, *b = Purse Seine*, *c = Danish Seines*, *d = Gillnets*, *e = Longlines, f = Traps/pots*, *N = Total sample size for respective FGs*

3 Similarly, every year fishing companies dispose end-of-life FGs from their stock and deliver it to either waste management facility or at the ports. This annual rate of FGs disposed of by fishing company is calculated for each FG type as an average of the ratio of reported FGs disposed of by a fisher from their respective stocks of FGs by coastal and ocean fishers.

Typical annual rates of gear disposal upon end-of-life (%)

$${C_{Disp}}_{\left(a,b,c,d,e,f\right)}=\frac{\sum_{n=1}^{114}{\left[\frac{{\left({FG}_{D}\right)}_{n}}{{\left({FG}_{O}\right)}_{n}}\right]}_{\left(a,b,c,d,e,f\right)}}{N_{\left(a,b,c,d,e,f\right)}}$$

*Where, a = Trawls*, *b = Purse Seine*, *c = Danish Seines*, *d = Gillnets*, *e = Longlines, f = Traps/pots*, *N = Total sample size for respective FGs*

## Method validation

The finalized questionnaire is then conducted among the commercial fishers in Norway to generate evidence on FG life cycle processes. The critical considerations while selecting the survey site, sample and mode of interaction with fishers are deliberated here to aid the effective implementation of the method and robust analysis of survey samples.

### Study area

**Fig. 3:**
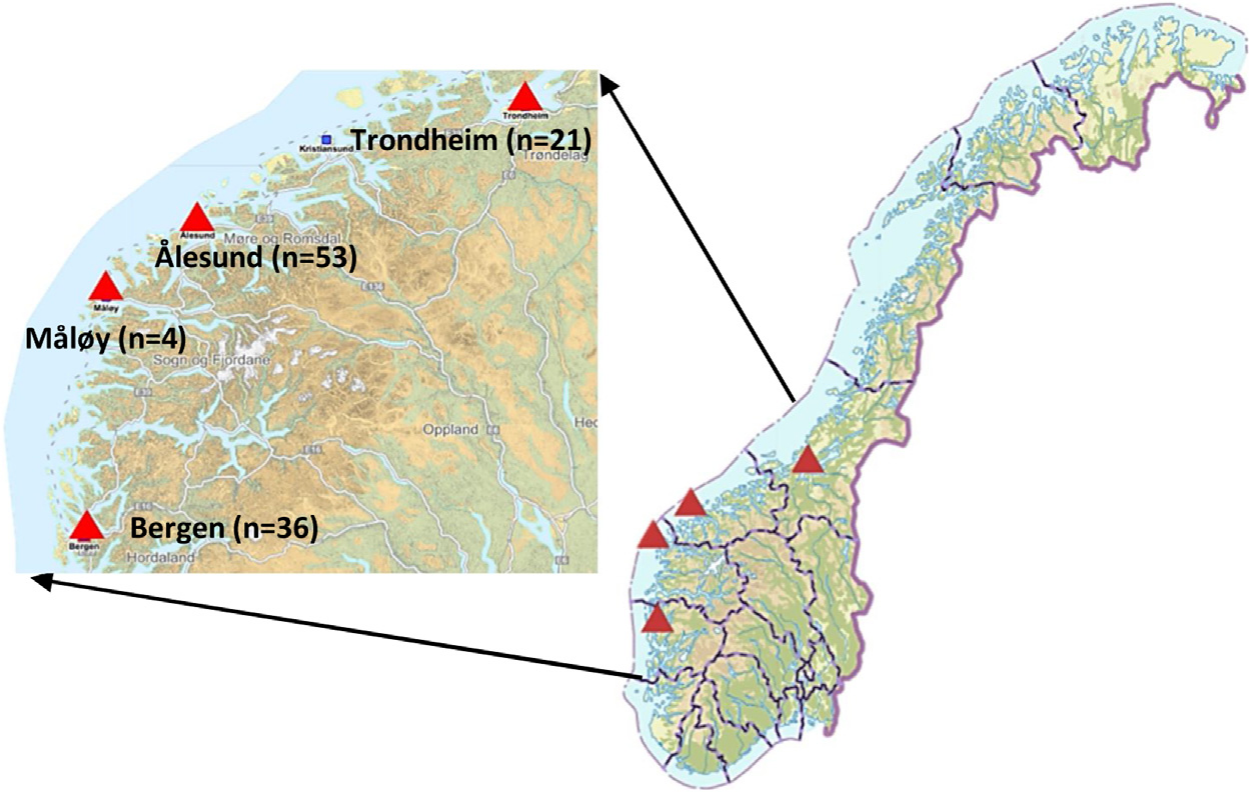
The map representing Norwegian coastline, fishing territory and selected four sampling locations to conduct questionnaire of local fishers between the period of August 2017 to January 2018 [28].

To avoid the bias and confusion in the responses, a face-to-face survey with fishers is preferred over an online questionnaire. Four, commercially important ports located on the west coast of Norway (Fig. 3) were chosen to interact with fishers. The selected sites namely, Bergen, Ålesund, Måløy, and Trondheim are home to both coastal and ocean fishing companies, FG suppliers and repair facilities. Moreover, these sites also host several fishery-related exhibitions, networking events and workshops for fishers, thereby, provides ample of opportunities to interact with fishers to conduct the desired questionnaire. To reach many fishers from diverse regions at the same time fishery-related exhibitions or conferences in the selected four study sites are targeted to conduct the questionnaire.

In total, 114 responses from fishers were collected in the span of 7-months from the selected sites. Fishers’ annual meetings, fishing product related conferences and exhibitions were targeted for conducting the survey. The collected sample responses were further analyzed using statistical methods to extract relevant information.

### Demographic and fishing characteristics of interviewed fishers

Commercial fishing practices vary with respect to demography, vessel size, target species and application of FGs. Therefore, it is essential to test the demographic characteristics of surveyed samples before analyzing the transfer coefficients. The response obtained from the questionnaire represents the well-distributed samples both regarding vessel size and the area of fishing activities. In total, 47% of the respondents belong to the coastal fishing fleet, and 53% represents a sophisticated and more massive ocean fishing fleet. Commercial fishing was the primary and full-time profession for most surveyed fishers, and this is consistent with the objectives of this study focusing on commercial fishing practices in Norway. Along the extensive coastlines of Norway, maximum commercial fishing takes place in northern, western and central parts of Norway, accordingly, the survey reflects more respondents fishing in the northern, western and central parts along with some minor fishing activities in eastern and southern parts. The demographic characteristics of survey samples are summarized in Table 1 to exemplify the representation of the surveyed samples.

**Table 1:** Demographic characteristics and fishing activity of the surveyed commercial fishing companies in Norway (N = 114).

Variables/Parameters	Coastal Fishing Fleet (V < 28 m)	Ocean Fishing Fleet (V > 28 m)
Number of Samples	55	59
**a) Occupation Level**		
• Full-time fishing	45 (86%)	57 (97%)
• Part time fishing	7 (14%)	2 (3%)
• Recreational	0	0
**b) Area of Fishing**		
• North Norway	30 (53%)	11 (19%)
• West Norway	15 (26%)	28 (49%)
• Mid Norway	07 (12%)	18 (32%)
• South Norway	05 (09%)	0 (0%)
**c) Type of FGs**		
• Trawls	06%	29%
• Purse Seines	06%	28%
• Danish Seines	14%	09%
• Gillnets	39%	17%
• Longlines	19%	17%
• Traps/Pots	16%	0
**d) Type of Fish Catch**		
• Pelagic Fish species	10%	36%
• Ground-fish species	80%	60%
• Crustaceans and mollusks species	10%	04%

The pattern and use of FGs depend on areas of gear deployment and type of target fish species. A coastal fishing fleet consisting of smaller vessels and use relatively cheap and less sophisticated FGs *namely*, gillnets, longlines and traps/pots. Less than 10% of the total surveyed coastal fishers reported using sophisticated FGs like trawls and purse seines. However, around 21% of the surveyed coastal fishers reported using Danish seines as a replacement to more sophisticated purse seine or trawls. Coastal fishers responded by using all the primary FGs depending on types of fish species they are catching. However, none of the fisher representing ocean fleet reported using traps/pots indicating the rare use of crab pots/traps by deep-water fishers. Ocean vessels generally perform deep-water fishing deploying advanced FG types such as trawls (pelagic, bottom and semi-pelagic), purse seines, Danish seines and multiple sets of gillnets and longlines. Trawls and seines are considered sophisticated/advanced gear types, as they are useful concerning both capacity and efficiency of catching the commercially important fish species. Application of these FGs is one of the underlying reason why the ocean fishing fleet is responsible for around 85% of total catch caught annually by Norwegian fishing fleet [21].

### Interpretation of transfer coefficient (TC)

After obtaining the desired and well-distributed sample size, statistical analysis of survey responses (step-8) is conducted to estimate the TCs. The defined TCs and their formulas are detailed in section 2.2. The summary of the sample statistical analysis of the TCs is presented in Table 2. This analysis results in quantifying the annual rates at which fishing fleet loses, repairs or disposes of listed FGs, the life span of gears, FGs present in stock and so on. This quantification can then be represented graphically to interpret the behavior of listed FG type across its system life cycle. This information can further aid decision making for the effective management of FG resources in the given region.

**Table 2:** Statistical analysis of parameters and estimation of TCs from the responses of commercial fishing companies in Norway.

TC	FG types	Sample size	Mean	Std. Dev	Std. Error	95% conf. interval
Life Span _(yrs)_	Trawls	31	2,8	1,8	0,3	0,6
	Purse seine	30	10,2	5,3	1,0	1,9
	Danish seine	20	3,9	1,8	0,4	0,8
	Gillnets	48	2,1	1,1	0,2	0,3
	Longlines	31	3,0	2,6	0,5	0,9
	Traps/pots	14	6,1	4,6	1,2	2,4

C_Disposal (% of owned stock)_	Trawls	31	25,1 %	23,6 %	4,2 %	8,3 %
	Purse seine	30	7,3 %	9,3 %	1,7 %	3,3 %
	Danish seine	20	11,4 %	8,4 %	1,9 %	3,7 %
	Gillnets	48	33,1 %	26,7 %	3,9 %	7,5 %
	Longlines	31	30,8 %	26,5 %	4,8 %	9,3 %
	Traps/pots	14	16,9 %	13,2 %	3,5 %	6,9 %

Similarly other TCs namely, C_Lost_ (% of owned stock); C_repair_, C_replace_, C_stock_ can be estimated as per calculation formula given in Section Statistical analysis.

These estimated rates show around Trawls have a life span of around 3 yrs. Moreover, C_dispose_ for Trawls shows that the fishing fleet reportedly disposes of around 25% of the total trawls owned by a fishing fleet every year. Similarly, annual rates at which fishers lose their FGs, fraction total FG type owned by the fishing company needing repair, a fraction of part being replaced during repair, the average life span of FGs can be calculated through survey responses.

## Fishers survey: lessons learnt

In this method article, we proposed a stepwise method for a questionnaire-based survey to extract information from fishers. Although FK is considered as a valuable and abundant source of information in the data-poor field, careful and systematic approach is required to extract vital information from fishers to minimize the bias and confusion. Once the objective of the study is established, much of the emphasis is given to developing the set of questions for the proposed questionnaire. The adopted Delphi method to revise the questions proved to be a useful technique to make the questions lucid, concise and apt with the help of experts in the field. Further, selection of questionnaire language is another critical choice as although many fishers understand the English language, use of local language (Norwegian) in conducting the survey is observed to be a more practical way to avoid confusion and to create a comfortable environment for the respondent.

Furthermore, in coherence with Leite and Gasalla [23], establishing the confidence of fishers is critical in transmitting their knowledge. Three strategies were implemented during the survey to achieve the fishers’ confidence, firstly, face-to-face interviews were conducted in most of the study locations and secondly, interviewers introduced themselves as a student with minimal knowledge in the fishery and demonstrated impartiality toward the issues addressed. Finally, surveyed fishers are also well-informed about the anonymity of the process. These three strategies resulted in more open and relaxed discussion with fishers. A friendly environment also resulted in gaining extra information alongside survey questions as many of the surveyed fishers took extra time in sharing their stories and issues in dealing with specific types of FGs along with the historical development of fishing practices in their community.

Although applied widely to extract local knowledge, survey as a method possess some constraints. One of the important one being responders being speculative while answering the specific questions where they lack knowledge. In the present study, the aim was to capture the patterns of FGs repaired, lost and disposed of annually. Accordingly, the presented questions demand summarizing the 10–20 yrs. of fishing practices for some respondents, and they may respond to such answers with a particular bias regarding their memory and report those incidents that hold specific importance to them instead of being objective. Additionally, conducting a face-to-face survey is both time consuming and expensive way of collecting responses. Alternatively, interactive online survey platforms can be explored if the survey questions are relatively simple and unambiguous.

In conclusion, surveying fishers provide an effective framework to extract FK that further assists in building evidence on parameters that are otherwise not measurable. These parameters can be used to estimate regional flows of plastic and other FG materials through material flow analysis (MFA) models. Furthermore, the simplicity of the stepwise method makes it practical and easily reproducible elsewhere to obtain the relevant scientific estimates on studied parameters for respective countries/regions, which is the critical necessity for good science.
