# Supplementary material for: A method to extract fishers’ knowledge (FK) to generate evidence for sustainable management of fishing gears
Source: MethodsX. 2019 May 8;6:1044–53. doi: 10.1016/j.mex.2019.05.008 (PMC6522690; doi:10.1016/j.mex.2019.05.008)
Supplement: Supplementary file 1 [file mmc1.pdf]

Survey of Norwegian Fishermen for the Circular Ocean Research Project

Section 1 - Introduction

Are you concerned about the health of our seas and oceans? If you are, please give us **10-minutes** of your time to answer this questionnaire. Your answers are kept **anonymous** and will really help us to find out how many fishing gears are currently **ghost fishing** in the ocean, and what can be done **to mitigate** this problem.

This questionnaire is **made for Norwegian fishermen and skippers**. The questions are formulated to collect the data of **single fishing vessel**. If you are working on several fishing vessels each year, report only about the vessel you work the most on.

Survey of Norwegian Fishermen for the Circular Ocean Research Project

Section 2 - Norwegian fishermen & fishing fleet

* 1. Are you a full-time or a part-time fisherman?

- ◯ Full-time
- ◯ Part-time

* 2. How long is the vessel you are working on?

- ◯ Under **10** meters
- ◯ Between **10** to **11** meters
- ◯ Between **11** to **15** meters
- ◯ Between **15** to **21** meters
- ◯ Between **21** to **28** meters
- ◯ **28** meters and above

* 3. In which Norwegian region is the vessel's main port located?

- ◯ North Norway
- ◯ Mid Norway
- ◯ West Norway
- ◯ South Norway
- ◯ East Norway

Map of the main Norwegian regions

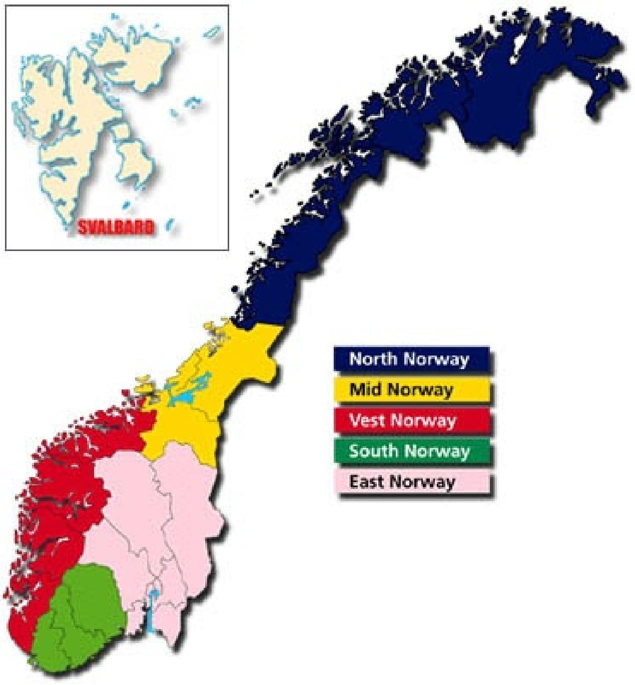

* 4. On average, which types of fish species are you fishing for?

Give an average percentage under each of the three categories (pelagic, groundfish, crustaceans).

The sum of the three percentages should be equal to 100%.

Example: 34% Pelagic species, 64% Groundfish species, 2% Crustaceans and molluscs.

Pelagic species [%] (Capelin, Norway pout, Blue whiting, Sandeels, Atlantic horse mackerel, Northeast Atlantic mackerel, Atlantic herring, Europan sprat)

Groundfish species [%] (Atlantic cod, Haddock, Saithe, Tusk, Blue ling, Greenland halibut, Atlantic redfishes, Argentines)

Crustaceans and molluscs [%] (Crab, Red king crab, European lobster, Norway lobster, Nothern prawn, molluscs, Antarctic krill)

Section 3 - Owned fishing gear

5. On average, how many of the following fishing gear types do you own at the same time?

If you don't use a type of gear, leave the category blank.

<u>Example</u>: 2 Trawls, 210 Gillnets, 35 Pots, etc.

Trawl

Purse Seine

Danish Seine

Gillnet

Longline

Trap/pot

* 6. On average, what percentages of gears owned are stored on board and stored at the port?

The sum of the two values must equal 100%.

<u>Example</u>: 37% on board / 63% at the port.

On board [%]

At the port [%]

Section 4 - Purchasing new fishing gear

7. On average, how frequently do you purchase the following fishing gear types?

Write a number and the corresponding time period under the following categories. If you don't use this type of gear, leave the category blank.

Example: 1 Purse Seine / 5-years, 1 Trawl / 1.5-years, 61 Gillnets / year, 21 Lonlines / year, etc.

Trawl	
Purse Seine	
Danish Seine	
Gillnet	
Longline	
Trap/Pot	

Survey of Norwegian Fishermen for the Circular Ocean Research Project

Section 5 - Repairing fishing gear

8. On average, how many percents of the following fishing gear types you own are being repaired each year?

The answer should **only include repairs involving the change of fishing gear parts**.

Write a percentage under the following categories. If you don't use this type of gear, leave the category blank.

Example: 12% Trawl, 18% Purse Seine, 6% Longline, etc.

Trawl [%]	
Purse Seine [%]	
Danish Seine [%]	
Gillnet [%]	
Longline [%]	
Trap/Pot [%]	

9. On average, how large are the parts replaced?

You should express the area of the part replaced as a **fraction of the whole fishing gear.**

Example: if most of the time the parts of trawls replaced represent approximately 15% of the whole trawl's area, you should select the box 10 to 20% for the trawl category.

	0 to 10%	10 to 20%	20 to 30%	30 to 40%	40 to 60%	I don't use this gear type
Trawl	○	○	○	○	○	○
Purse Seine	○	○	○	○	○	○
Danish Seine	○	○	○	○	○	○
Gillnet	○	○	○	○	○	○
Longline	○	○	○	○	○	○
Trap/Pot	○	○	○	○	○	○

10. On average, how do you spend the vessel's yearly fishing gear budget?

The objective is to determine which percentage of the fishing gear budget is used to buy new fishing gears and which percentage is used to repair damaged ones.

Example: new equipment = 59% / repair damaged equipment = 41%

Percentage used to buy new equipment [%] ____________________

Percentage used to repair damaged equipment [%] ____________________

Survey of Norwegian Fishermen for the Circular Ocean Research Project

Section 7 - Fishing gear lifespan

11. On average, what is the lifespan of the following fishing gear types?

Write a number of years under the following categories. If you don't use this type of gear, leave the category blank. In this case, we are assuming the gears are used until they are too worn to be repaired. **Do not account for gear losses when evaluating fishing gear lifespan**.

Trawl	
Purse Seine	
Danish Seine	
Gillnet	
Longline	
Trap/Pot	

Section 8 - Fishing gear lost at sea

12. On average, how frequently do you lose the following fishing gears at sea?

Write a **number and the corresponding time period** under the following categories. If you don't use this type of gear, leave the category blank. This question is focusing on the loss of whole fishing gears only. **Do not include the loss of fishing gear parts in this question.**

Example: 1 Trawl / 5-years, 1 Purse Seine / 4-years, 15 Gillnets / year, 25 Pots / year, etc.

Trawl

Purse Seine

Danish Seine

Gillnet

Longline

Trap/Pot

Survey of Norwegian Fishermen for the Circular Ocean Research Project

Section 9 - Fishing gear end-of-life

13. On average, how many percents of the following fishing gear types you own needs to be disposed of annually?

Write a percentage under the following categories. If you don't use this type of gear, leave the category blank.

Example: 11% Danish Seine, 26% Gillnet, 7% Trap/Pot, etc.

Trawl [%]

Purse Seine [%]

Danish Seine [%]

Gillnet [%]

Longline [%]

Trap/Pot [%]

* 14. Where do you deliver the fishing gears you want to dispose of?

Write the name of the company/facility/place collecting this waste.

Thank you for your participation!

Contacts

Paritosh Deshpande (paritosh.deshpande@ntnu.no)
Gaspard Philis (gaspard.philis@ntnu.no)
